# Degradation Behavior of Poly(Lactide-Co-Glycolide) Monolayers Investigated by Langmuir Technique: Accelerating Effect

**DOI:** 10.3390/molecules28124810

**Published:** 2023-06-16

**Authors:** Gayeon Kim, Vishal Gavande, Vasi Shaikh, Won-Ki Lee

**Affiliations:** 1 Department of Polymer Engineering, Pukyong National University, Busan 48513, Republic of Korea; gayeon203@naver.com (G.K.); vgawande77@gmail.com (V.G.); 2 Hankuk Paper Mfg. Co., Ltd., Ulsan 45010, Republic of Korea; 3 Department of Chemistry, Dr. Vishwanath Karad MIT World Peace University, Pune 411038, India; vasi.shaikh@mitwpu.edu.in

**Keywords:** poly(lactide-co-glycolide) (PLGA) copolymers, copolymerization, alkaline hydrolysis, enzymatic degradation, Langmuir monolayers

## Abstract

Among biodegradable polymers, polylactides (PLAs) have attracted considerable interest because the monomer can be produced from renewable resources. Since their initial degradability strongly affects commercial application fields, it is necessary to manage the degradation properties of PLAs to make them more commercially attractive. To control their degradability, poly(lactide-co-glycolide) (PLGA) copolymers of glycolide and isomer lactides (LAs) were synthesized, and their enzymatic and alkaline degradation rates of PLGA monolayers as functions of glycolide acid (GA) composition were systematically investigated by the Langmuir technique. The results showed that the alkaline and enzymatic degradations of PLGA monolayers were faster than those of l-polylactide (l-PLA), even though proteinase K is selectively effective in the l-lactide (l-LA) unit. Alkaline hydrolysis was strongly affected by their hydrophilicity, while the surface pressure of monolayers for enzymatic degradations was a major factor.

## 1. Introduction

Petroleum-based polymers are widely used because of their favorable properties as durable, lightweight, and inexpensive materials. However, huge amounts of waste from non-degradable and disposable plastics cause serious long-term environmental problems for plants, animals, and humans. Overall, in the USA, the amount of recycled plastics is relatively small—three million tons at an 8.7 percent recycling rate in 2018 (the United States Environmental Protection Agency) [1]. Most end up in landfills or incineration. Some discarded plastics are recognized as a severe anthropogenic issue in coastal and marine ecosystems. Additionally, plastics made from fossil fuels require a lot of energy for synthesis and processing, which results in global warming [2,3,4]. To minimize these issues, degradable and renewably derived polymers are significantly utilized in the fields of biomedical and commercial applications. The most widely researched biodegradable polymers are polylactide (PLA), poly(butylene adipate terephthalate) (PBAT), polyglycolide (PGA), poly(e-caprolactone) (PCL), and poly(butylene succinate) (PBS), which have nontoxic and biocompatible properties [5,6,7,8]. These biodegradable polyesters are usually degraded by the enzymatic or hydrolytic degradation, or both, of an ester bond, leading to low-molecular-weight oligomers, dimers, and monomers, and, finally, are mineralized to CO_2_ and H_2_O [5,6,7,8,9,10]. However, it is imperative to control the degradation rate of degradable polymers for pharmaceutical or commercial applications. A fast and constant hydrolytic rate of drug-loaded biopolymers is required in drug-delivery systems. However, the 1–2% weight loss of biopolyesters by degradation dramatically affects the service life of commercialized items because of the 66% loss of its mechanical property [9,10]. Accordingly, the mechanical properties of biopolyesters are rapidly lost during the initial degradation. An ideal industrial good is stable during use and quickly degraded when discarded. Therefore, tuning the degradation rate of biopolymers during their applications is receiving more consideration, and it can be regulated by chemical or physical modifications such as copolymerization, blending, and surface treatments [11,12,13]. 

PLA homopolymers exhibit a glass transition temperature (T_g_) ranging from 50 °C to 80 °C, and they undergo complete biodegradation in approximately ten months through the hydrolysis of ester bonds in the polymer backbone. The degradation process can be influenced by various factors such as temperature and pH or the presence of enzymes and moisture. PLA typically degrades slowly in ambient conditions, but the degradation rate can be accelerated in composting environments or under controlled conditions in industrial composting facilities. However, the hydrophobic nature of PLA can restrict its biomedical applications due to low water sorption. On the other hand, PLA’s high crystallinity imparts exceptional mechanical properties. PGA with a T_g_ of around 35 °C has a similar structure to PLA but different degradability and physical and mechanical properties due to the absence of a methyl group on the alpha carbon [14]. PGA is more susceptible to degradation than PLA due to its higher hydrophilicity. The hydrolysis of PGA results in the formation of glycolic acid and its oligomers. PGA, on the other hand, experiences full enzymatic biodegradation within four months, but its mechanical properties deteriorate significantly after just six weeks [15,16,17]. PGA degrades faster than PLA and has been widely used in biomedical applications where the material is intended to be absorbed by the body. It is important to note that both PLA and PGA can be tailored to have different degradation rates and properties by adjusting their molecular weight and composition, incorporating additives or modifiers, or copolymerization. Overall, the degradation of PLA and PGA offers the advantage of reducing environmental impact and providing biocompatible alternatives to traditional non-degradable plastics in various applications. Furthermore, the use of PGA itself is limited by its hydrolytic instability and insolubility in most organic solvents. Therefore, it is widely copolymerized with LA or CL, which have a relatively slow degradation rate, when applied in the field of drug-delivery polymers as nanocarriers, sutures, and implants and in medicine as an absorbent material. The application of poly(lactide-co-glycolide) (PLGA) in the pharmaceutical industry has increased in numerous drug-delivery formulations due to its biocompatibility, biodegradability, and tunable degradation properties. Additionally, it is possible to control the solubility and thermal properties of the PLGA copolymer by changing the lactide/glycolide ratios, molecular weight, and end-cap functional groups of the starting material [16,17,18].

PLGA has become widespread in the biomedical field, and it is easily broken down in vivo by hydrolysis into lactic acid and glycolic acid. Although there have been numerous studies on the degradation of PLGA, very few studies have been conducted at the molecular level [17,18,19,20]. It is important to analyze the initial degradation behavior because the degradation starts with the very thin surface layer of the biopolyester. The Langmuir technique is one of the most suitable techniques to study the hydrolytic behavior of polyesters at monolayer levels since most polyesters can form monolayers owing to their hydrophilic/hydrophobic balance [21]. Biopolyesters can undergo hydrolytic scission in their main chains at a constant surface pressure, and the cleavage of ester groups eventually produces water-soluble fragments. Their dissolution into subphase leads to a reduction in the occupied area as a function of time, which demonstrates a hydrolytic kinetic at the molecular level. Moreover, Langmuir monolayers stipulate a fast and attractive path compared to bulk materials [22]. In this study, PLGA monolayer behavior in air/water was investigated with different degradation mediums, alkaline and enzymatic, and surface pressures. This study aimed to collate research on optimizing the synthesis procedure by changing parameters, such as the lactide/glycolide ratios, that can affect degradation behaviors at the molecular level; therefore, a great deal of deliberation went into designing the system for specific implementation, and it can be expanded to a broader range of commercial applications. 

## 2. Results and Discussions

### 2.1. Synthesis of Copolymers

**Figure 1:**
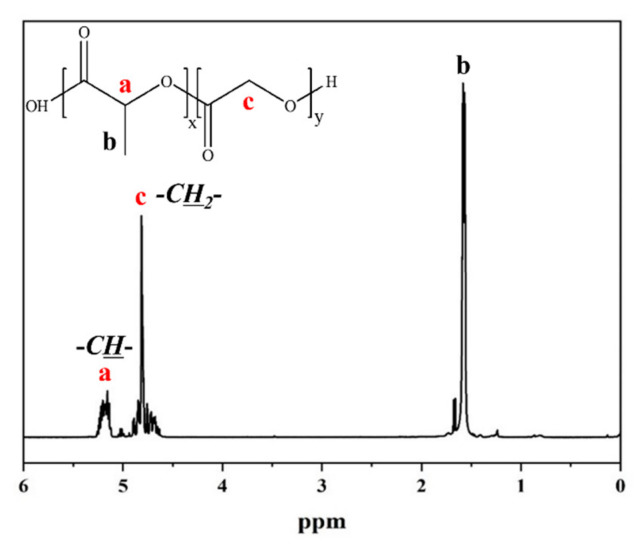
^1^H-NMR spectrum of the PLGA copolymer and its structure (a and b peaks attributed to LA, while c peak was assigned to GA, confirming the successful synthesis of the copolymer).

Figure 1 depicts the ^1^H-NMR spectra of the PLGA copolymers. It represents those copolymers that were successfully synthesized through the peaks corresponding to the methyl group -CH- protons (peak at 5.2) of the LA and the methylene group -CH_2_- protons (peak at 4.8) of the GA. The compositions of synthesized PLGAs were confirmed by the integral of these two peaks. The detailed compositions of the copolymers used in this study are listed in Table 1. In other experiments in the literature [15,16,17], the composition of the GA in PLGA was higher than that of the in-feed monomer because GA has higher reactivity than LA in ring-opening polymerization. However, the solution polymerization method applied in this experiment did not show a similar tendency because the monomers differed appreciably in solubility and diffusion rate when comparing the bulk polymerization. Generally, the same reactivity ratios apply whether the polymerization is carried out in a bulk, solution, suspension, or emulsion system. Therefore, deviations in copolymer composition can only be expected from concentration differences in polymerization sites [23]. Factors such as monomer concentration gradients, differences in solubility, and variations in monomer diffusion rates can affect the local monomer concentrations within the polymerization system. These deviations can lead to variations in the copolymer composition, particularly in systems with uneven distribution or concentration gradients of monomers.

**Figure 2:**
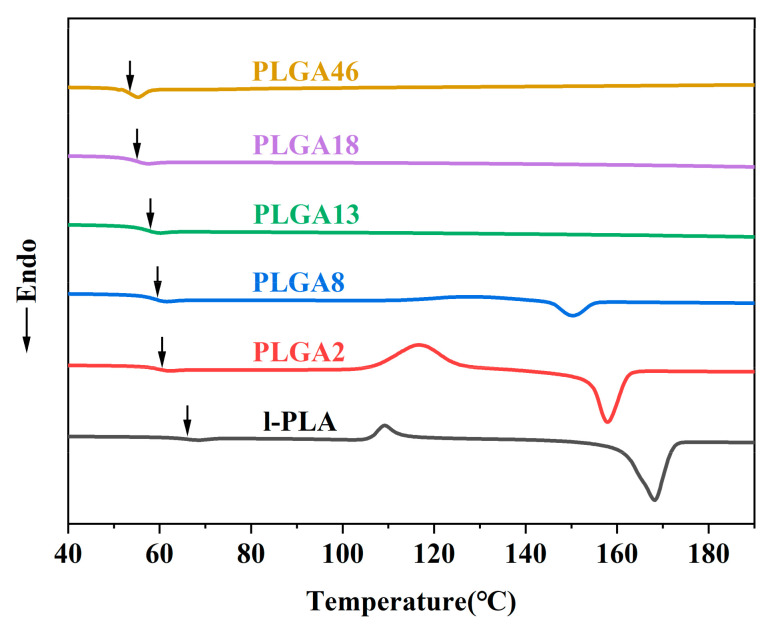
DSC curves of synthesized polymers. The arrows represent the T_g_ taken as the middle point.

Figure 2 represents the thermal properties of the synthesized copolymers with different GA contents. It was observed that minor incorporation of GA in LA chains decreased the T_m_ and crystallinity because GA units interrupted the chain regularity of long l-LA chains (Table 2). Therefore, this conformation deteriorated the perfectness of the crystal. The representative DSC thermograms illustrated that the decrease in the crystallinity of the synthesized copolymers decreased with increasing GA contents due to the random structure. Additionally, the copolymers showed a single T_g_, which decreased with increasing GA content. It was evidenced that PLGA copolymers ≥ 13 mol% GA were amorphous and exhibited a single T_g_ without an endothermic peak [21], and this thermal behavior implies that the PLGA copolymers were successfully synthesized. The detailed thermal properties and molecular weights of the synthesized PLGA copolymers are summarized in Table 2.

### 2.2. Surface Pressure–Area Isotherms

The Langmuir system can be applied to make a monolayer of biodegradable polyesters attributable to their hydrophilic/hydrophobic balance. Surface pressure–area (π-A) isotherms have a mammoth advantage when studying the polymer monolayers on a subphase. This surface pressure is defined as the differentiation between the surface tension of the subphase and the surface tension in the presence of a monolayer of the material, which is measured by the Wilhelmy method [11,24]. The particular orientation and packing of molecules on the subphase correspond to a specific state. With increasing surface pressure, the molecules approach each other, and their packing density increases. Some monolayers show a plateau region in the isotherm, which means a transition of phase and formation of a three-dimensional structure and conformation changes [25]. 

**Figure 3:**
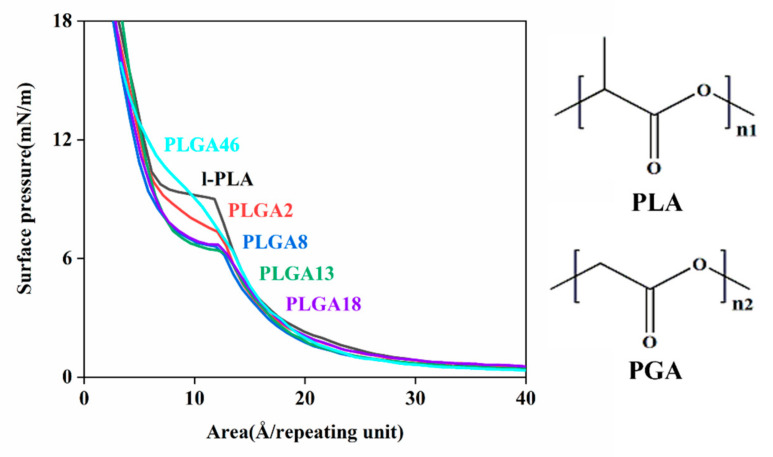
Pressure–area isotherms of l-PLA and PLGA monolayers on subphase of deionized water.

Figure 3 shows the π-A isotherms of the l-PLA and PLGA monolayers on deionized water. The flat transition region of l-PLA was observed at 9.2 mN/m, and it was changed proportionally as the GA content in copolymers increased. Although LA has an additional pendent methyl group compared to GA, all π-A isotherms are similar to each other except for in transition regions. The flat transition of l-PLA was changed to a sloped transition with GA contents at lower surface pressure, and, in the PLGA 46 monolayer, there was no transition-like behavior. This behavior would have been regularity of polymeric chains. As shown in the DSC data, the crystallinity of the copolymers decreased with GA content, and the transition behavior of the PLGA monolayer was strongly related to their unit regularity. This was supported by the π-A isotherm of dl-PLA, which was in the amorphous region and did not show any transition behavior [21].

### 2.3. Alkaline Hydrolysis of Monolayers

**Figure 4:**
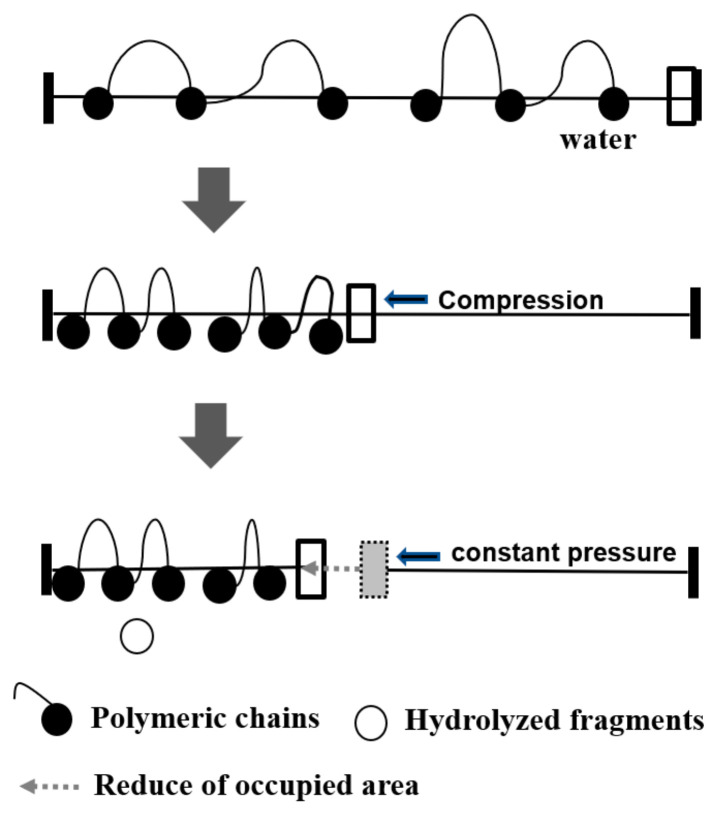
Schematic representation of hydrolyzable polymer monolayers on the water subphase.

The Langmuir system allows a fast and elaborate route to determine the hydrolytic kinetics of biodegradable polymers at a molecular level [4,5,7,26]. The state of the biodegradable polymer monolayer was monitored and analyzed on a Langmuir trough (Figure 4). To study the hydrolytic degradation of monolayers, a dilute polymer solution with a volatile solvent was spread on a subphase. After compressing the monolayers by the barrier to a desired surface pressure, the occupied area of the polymeric chains at a constant surface area was changed with the degradation time since the hydrolysis of biopolymers produced a water-soluble by-product. Therefore, the A/A_0_ value with time reflected the hydrolytic rate, where A and A_0_ denote the occupied areas at times t and 0, respectively. The degradation medium in a subphase can be controlled by the concentration of enzymes and active ions.

**Figure 5:**
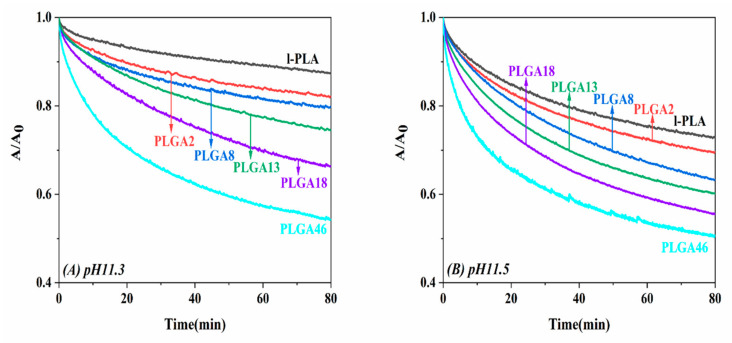
Area ratio vs. time for l-PLA and PLGA monolayers maintained at 5 mN/m on subphase pH 11.3 (**A**) and pH 11.5 (**B**).

Firstly, the effects of subphase pHs (ion concentration) 11.3 and 11.5 on the hydrolytic behavior of monolayers were studied at a constant surface pressure of 5 mN/m. Although the M_n_ of l-PLA and PLGAs was similar, the alkaline hydrolysis rate increased with the GA content in the copolymer (Figure 5). Generally, alkaline hydrolysis of biodegradable polyesters is strongly affected by their hydrophilicity because the water solubility of fragments produced by the hydrolysis increases [7,27,28,29,30]. The water contact angles of the l-PLA and PLGA46 cast films were 72° and 66°, respectively. Therefore, the monolayer was more submerged in subphase as the GA content in the copolymer increased, and it provided more hydrolyzable sites for sodium ion attack. Moreover, the low steric hindrance of GA due to the absence of the methylene group was easier than LA for hydroxide ions to attack. When increasing subphase pH from 11.3 to 11.5, all hydrolytic rates increased. At subphase pH 11.5, l-PLA showed over two times faster hydrolysis, whereas PLGA46 increased very little. A noticeable point is that PLGA46 had very similar hydrolytic kinetic behaviors, regardless of pH. For a better understanding of the hydrolytic behavior of monolayers in an alkaline subphase, additional experiments were carried out with different pHs with a constant pressure of 5 mN/m. 

**Figure 6:**
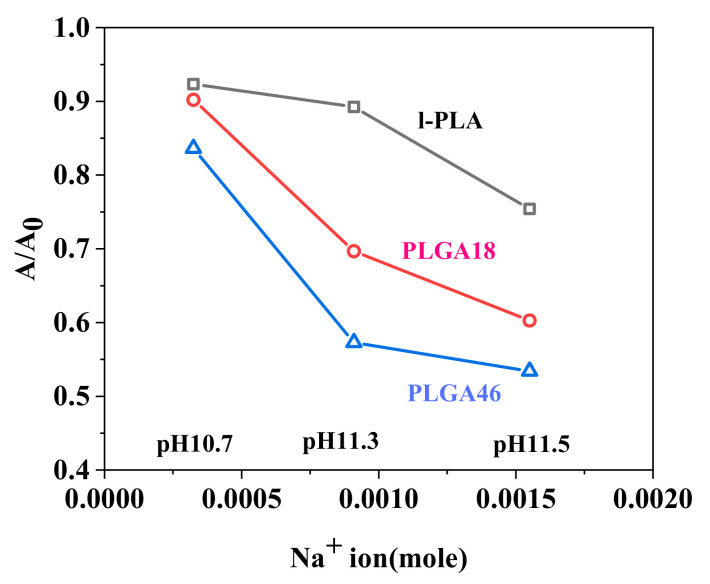
Area ratio vs. pHs for monolayers maintained at 5 mN/m when the hydrolytic time is 60 min.

Figure 6 shows the A/A_0_ values of l-PLA, PLGA18, and PLGA46 monolayers at 5 mN/m after 60 min on different pHs. The hydrolysis rate of the monolayers increased with GA content in the polymer regardless of subphase pH. The hydrolysis of the l-PLA monolayer was relatively slow, and its hydrolytic rate increased much more rapidly with increasing pH. However, the hydrolytic rate of PLGA monolayers rapidly increased from pH 10.7 to 11.3 and then their hydrolytic rates were slow. Concentrations of Na^+^ ions were about 1, 2, and 4 times greater for pH 10.7, 11.3, and 11.7, respectively. From the results of this alone, it can be considered that the critical point of the amounts of ions depends on the hydrophilicity of the monolayers, which is related to submerged chain length and solubility to subphase.

**Figure 7:**
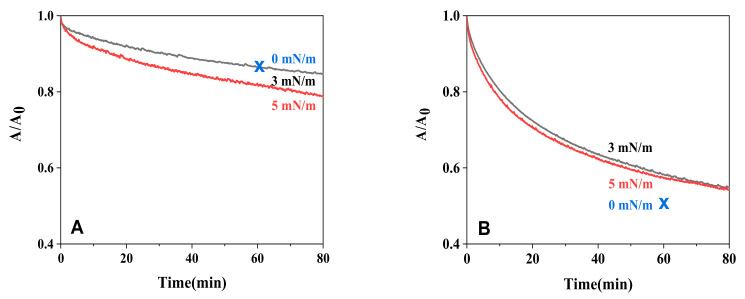
Area vs. time for l-PLA (**A**) and PLGA46 (**B**) monolayers on the subphase of pH 11.3. Area ratio at 0 mN/m was calculated at 0 and 60 min of being held from spreading.

Figure 7 shows a plot of the A/A_0_ vs. time of l-PLA and PLGA46 monolayers at various constant surface pressures at pH 11.3, and X in the figure represents the occupied area after 60 min of holding time from spreading. The hydrolytic rate of l-PLA monolayers showed a slight increase with surface pressure. At 0 mN/m, the occupied area of the monolayer was larger, while submerged monolayers in the subphase were smaller. Therefore, hydrolysis of l-PLA monolayers at 0 mN/m slowly occurred, while the monolayers at 5 mN/m showed a relatively fast hydrolytic rate due to the increase in submerged monolayers. In the PLGA46 monolayers, however, the extent of the hydrolysis of the PLGA46 monolayers followed the order 0 > 5 ≥ 3 mN/m. This result indicates that the occupied area of PLGA46 monolayers was more affected than the surface pressure. Therefore, it was found that the area in contact with the ions and the hydrophilicity play an important role in alkaline hydrolysis.

### 2.4. Enzymatic Degradation of Monolayers

**Figure 8:**
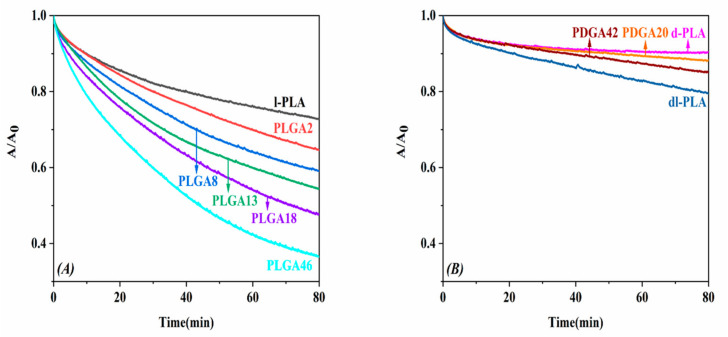
Area ratio vs. time for (**A**) l-PLA and PLGA copolymers, and (**B**) d-PLA,dl-PLA, and PDGA copolymers monolayer films maintained at 5 mN/m on subphase with 0.02 mg of proteinase K.

The Langmuir technique was shown to be a phenomenal technique for acquiring insights into the interaction between polymer chains and enzymes. It was proved that enzymes require a minimum packing density of the polymeric substrate to become active [26]. Proteinase K can catalyze the degradation of l-PLA in amorphous regions, although the folded chains in the crystalline region are highly stable to enzymatic cleavage, while PGA is known to degrade specific enzymes, especially exoenzymes such as lipase, esterase, and endopeptidase [31,32]. The enzymatic degradation of l-PLA and PLGA monolayers was examined at 5 mN/m on the subphase with 0.02 mg of proteinase K, as shown in Figure 8. The enzymatic degradation rate of PLGA increased with GA composition, and PLGA46 was shown to have the fastest degradation rate.

It is known that proteinase K can catalyze the degradation of l-LA units but cannot catalyze the degradation of d-LA ones [12,27,28,33]. As shown in Figure 8B, the dl-PLA containing 20 mol% of d-LA showed a delay in degradation rate even though it was amorphous. Although PLGA18 and PLGA46 were equally amorphous, their monolayers showed faster enzymatic degradation than the l-PLA one. We established two possible assumptions: (i) the GA showed activity on proteinase K, and (ii) the GA accelerated the solubility of fragments. To prove this, we synthesized two PDGA copolymers containing 20 mol% and 42 mol% compositions of GA and carried out enzymatic degradation experiments. The d-PLA, PDGA20, and PDGA42 were approximately 90% (A/A_0_ = 0.90), 88% (A/A_0_ = 0.88), and 85% (A/A_0_ = 0.85), respectively, after 80 min of degradation. These results evidenced that proteinase K did not catalyze the GA. As mentioned above, as the GA units increased in the copolymer, the monolayer was more submerged in the subphase due to its hydrophilicity, and it helped to be attacked by the enzymes that selectively degraded l-LA [6,33]. Other studies used the term “catalytic effect” because GA plays a role in promoting the degradation of PLA, and this tendency was also observed in the Langmuir system [28,33]. 

**Figure 9:**
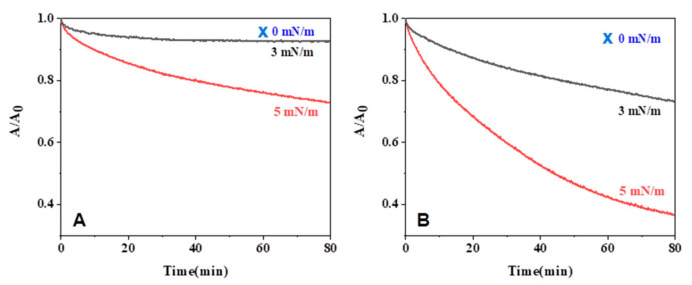
Area ratio vs. time for l-PLA (**A**) and PLGA46 (**B**) monolayers on the subphase with 0.02 mg of proteinase K at different constant surface pressures.

**Figure 10:**
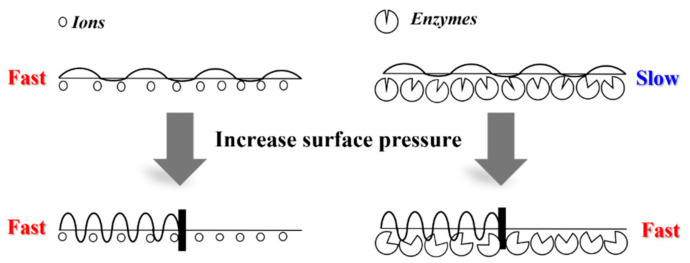
Schematic representations for alkaline and enzymatic degradations of biodegradable polyester monolayers.

To investigate the effect of surface pressure on enzymatic degradation behavior compared to alkaline hydrolysis behavior, the monolayers were preceded at various surface pressures and 60 min of hold time (0 mN/m). As shown in Figure 9, enzymatic degradation showed different behaviors compared to alkaline hydrolysis. The monolayers were hardly degraded after 60 min of hold time, but the reduction of the occupied area was faster as the surface pressure increased. At a high surface pressure, polymeric chains are more submerged into a subphase due to the increase of their density, and their hydrophilicity also increases the submerged part into a subphase. This trend is related to the comparative size of alkaline ions and proteinase K; the Na^+^OH^−^ ions are much smaller than proteinase K (ca. 29 kDa) and can hydrolyze the monolayers at a low constant surface pressure [7]. In contrast, proteinase K can be activated above high constant surface pressure. Therefore, the activity increases due to the wide contact of monolayers with enzymes in the subphase at high constant surface pressure. We hypothesize that the PLGAs monolayers are seriously affected by the constant surface pressure in enzymatic degradation, unlike in alkaline hydrolysis (Figure 10). 

## 3. Experimental

### 3.1. Materials

L- and d-lactide (d-LA) were procured from Purac (Gorinchem, The Netherlands), while glycolide and tin(II) 2-ethylhexanoate were obtained from Sigma-Aldrich (St. Louis, MO, USA). Chloroform, toluene, and methanol were sourced from Samchun (Seoul, Republic of Korea). All chemicals used were of reagent grade and did not require additional purification.

### 3.2. Synthesis of PLGA

PLGA was synthesized by the ring-opening bulk polymerization of LA and GA monomer with the presence of tin(II) 2-ethylhexanoate as a catalyst. A weighed amount of LA and GA and two wt% of catalyst were added to the polymerization vessel, and the experiment was carried out in a vacuum-sealed vessel at 140 °C for 6 h. Although this bulk polymerization method is simple and widely used in industries at the commercial scale to obtain a high-molecular-weight polymer, it is very challenging to control the molecular weight because of the automatic acceleration reaction in the absence of solvents. However, with the solution polymerization method, it is easier to control the molecular weight polydispersity index (PDI) by adjusting the reaction time, temperature, amount of solvent, and catalyst concentration. Many experimental conditions and variables were changed to synthesize polymers of the desired molecular weight. 

To conduct solution polymerization, 5 g of LA and GA was introduced into a round-bottom flask of 250 mL capacity equipped with a condenser. Toluene was preferred as the solvent for polymerization due to its low chain transfer constant value and high boiling point of 110.6 °C. The molecular weight of the final polymer can be influenced by the chain transfer constant of the solvent used, which can cause a reduction in the degree of polymerization. Briefly, specific quantities of lactide and glycolide, along with 500 μL of tin(II) 2-ethylhexanoate, were added to 15 mL of toluene. The mixture was heated in an oil bath to 110 °C and left to react for 24 h. To eliminate any remaining unreacted monomers, the resulting product was dissolved in chloroform and precipitated in an excess of methanol. The final product was filtered, and any remaining moisture was removed through vacuum desiccation.

dl-PLA and PDGA were prepared through solution polymerization using l-LA and d-LA monomers and d-LA and GA monomers, respectively. The polymerization reaction was facilitated by the presence of tin(II) 2-ethylhexanoate as a catalyst. The same procedure as mentioned above was used for the synthesis. However, if the composition of glycolide in the product exceeded 20 mol%, it was highly crystalline and unable to dissolve in toluene. In this case, bulk polymerization was utilized to synthesize PLGA (or PDGA) with a glycolide content of over 20 mol%.

### 3.3. Characterization of PLGA Copolymer

Gel permeation chromatography (GPC) was employed to determine the number average molecular weight (Mn) and PDI of the samples. The analysis was conducted using a Shimadzu Corp GPC system in chloroform at a flow rate of 0.8 mL/min with polystyrenes (Shodex^®^ STANDARD SM-105, Showa Denko K.K., Tokyo, Japan) used as reference standards. The composition of the copolymers was identified with a Fourier-transform nuclear magnetic resonance spectrometer (FT-NMR, JEOL, JNM ECZ-400) with CDCl_3_ as the solvent. The thermal properties were determined at a heating rate of 10 °C/min and a cooling rate of −10 °C/min by differential scanning calorimetry (DSC 1, Mettler Toledo, Columbus, OH, USA) under a nitrogen atmosphere. 

For the investigation of monolayer properties, a computer-controlled KSV-NIMA Langmuir probe system was utilized. The trough size used was 580 × 145 × 4 mm^3^. All solutions were prepared separately at a concentration of 1 μmol/mL. The subphase liquid consisted of approximately 450 mL of deionized water (DI, 18.2 MΩ·cm) purified by a Sinhan Science (Daejon, Republic of Korea) ultrapure water system. Prior to compression, the solvent was allowed to evaporate for 1 min to minimize hydrolysis. The barriers were compressed at a rate of 10 cm^2^/min in all experiments.

### 3.4. Degradation Behavior of Monolayers

For the alkaline hydrolysis of the monolayers, NaOH (Icatayama Chemicals, Tokyo, Japan) was added to the subphase to maintain the desired pH. On the other hand, enzymatic degradation of the monolayers was conducted using proteinase K obtained from Tritirachium album (Sigma-Aldrich) in a 25 mM Tris-HCl buffer with a pH of 8.6. The pH of the buffer solution was adjusted using HCl solution (Samchun, Seoul, Republic of Korea), and the pH of all solutions was measured using a pH meter equipped with an electrode.

## 4. Conclusions

The Langmuir technique was employed to investigate the degradation behavior of PLGA monolayers under alkaline and enzymatic conditions while maintaining a constant surface pressure. 

Alkaline hydrolysis was strongly affected by the occupied area and the hydrophilicity of monolayers. Hydrolysis of monolayers with strong hydrophilicity was accelerated by increasing an occupied area, while weak hydrophilic monolayers showed fast hydrolysis by increasing the constant surface pressure. This indicates that the hydrophilicity of monolayers is related to submerged parts of the polymeric chain in the subphases in which hydrolysis occurs. On the other hand, enzymatic degradation occurs at high surface pressure, as more contact area between enzymes and submerged chains is necessary. The degradation rate was found to increase with higher GA composition, despite GA being inactive for proteinase K. This is likely due to the many hydrophilic GA units in hydrolyzed fragments increasing their solubility into subphase. This was proved by more corresponding evidence obtained by synthesizing d-PLA and two different molecular weights of PDGA for enzymatic degradation. The d-PLA, PDGA20, and PDGA42 were approximately 90% (A/A_0_ = 0.90), 88% (A/A_0_ = 0.88), and 85% (A/A_0_ = 0.85), respectively, after 80 min of degradation. Overall, the study suggests that alkaline hydrolysis occurs in the small contact area between monolayers and subphase ions, while enzymatic degradation requires large contact areas. This is supported by alkaline hydrolysis occurring in both the crystalline and amorphous regions, whereas enzymatic degradation preferentially occurs in the amorphous region. By changing the surface pressure and degradation medium of biodegradable polyester monolayers, it is possible to understand the degradation behavior in a short time and use it as basic data for the commercial design of new biodegradable polymers.

## Figures and Tables

**Table 1:** Composition of materials used in this study.

Sample Code	In Feed (mol%)		Product (mol% by NMR)	
	l-LA	GA	LA	GA
l-PLA	100	0	100	0
PLGA2	95	5	98	2
PLGA8	90	10	92	8
PLGA13	85	15	87	13
PLGA18	80	20	82	18
PLGA46 *	70	30	54	46
PDGA20 **	80	20	80	20
PDGA42 *^,^ **	70	30	58	42

* Bulk polymerization. ** d-LA instead of l-LA.

**Table 2:** Characterization of polymers used in this study.

	M_n_	PDI	T_g_	T_m_	Crystallinity (%)
l-PLA	28K	1.6	64.2	169.6	44.7
PLGA2	27K	2.3	60.5	158.2	4.4
PLGA8	27K	2.3	59.3	150.3	2.8
PLGA13	26K	2.4	58.0	-	
PLGA18	16K	3.8	54.9	-	
PLGA46	23K	2.1	53.6	-	
PDGA20	28K	2.2	55.0	-	
PDGA42	-	-	49.83	-	

## Data Availability

Not applicable.

## Sample Availability

Not available.
